# A Truly Broad View of Gene Expression Spotlights Evolution and Diversity

**DOI:** 10.1371/journal.pbio.0020018

**Published:** 2003-12-15

Bioinformatics and microarrays have given scientists powerful new tools to investigate the structure and activity of genes on a global scale. Rather than studying just a few genes, scientists can analyze tens of thousands within and across species. Microarrays flag which genes are expressed under particular cellular conditions in an organism, while genome sequencing offers clues to gene function and regulation. By comparing the genomic properties of different species, scientists can spot patterns that help them identify functional and regulatory elements, learn about genome structure and organization, and gain a better understanding of the evolutionary forces that shape life on Earth.

The potential of these technologies to reveal insights into the fundamental structure and function of biological systems continues to grow along with the wealth of gene sequence and expression data—but the ability to interpret and merge these datasets lags behind the ability to collect them. In an effort to overcome these limitations, Sven Bergmann, Jan Ihmels, and Naama Barkai developed a comparative model that integrates gene expression data with genomic sequence information.

Because functionally related genes are expected to be coexpressed in different organisms and because the sequence of some of these functionally related genes may also be conserved between organisms, Bergmann and colleagues hypothesized that “conserved coexpression” could serve as an indicator of gene function on a genomic level. (Conserved genes are those that have changed little since they first evolved. Conserved coexpression describes functionally related genes that are activated together in different species.) But first they had to determine whether coexpression was conserved among species. Analyzing the gene expression profiles of six distantly related organisms—bacteria, yeast, plant, worm, fruitfly, and human—the researchers found that functionally related genes were indeed coexpressed in each species. The most strongly conserved sets of coexpressed genes are associated with core cellular processes or organelles. These results indicate that conserved coexpression can improve the interpretation of genome sequence data by providing another functional indicator for homologous sequences.

Since functionally related genes are expressed together in different organisms, it would be reasonable to think their regulatory networks are also conserved. To explore this idea, the researchers grouped coexpressed genes and their regulatory elements into “transcription modules” for each organism. They found significant variation in the number, organization, and relative importance of these modular components. Which components contributed most to an organism's global transcription program, for example, depended on the organism. But they also found that the transcription networks are highly clustered—meaning that genes connected to a specific gene are also connected to each other. This finding indicates that gene expression programs, regardless of their size or individual components, are highly modular. Each transcriptome contains modules that have been conserved over time along with “add-on” modules that reflect the needs of a particular species. This modularity supports the notion that variation between and among species arises from the diversity of gene expression programs.

Although the regulatory details of individual gene groups varied, the researchers found common ground in the overall landscape of the expression data. The transcription programs exhibit properties typical of dynamically evolving “real-world” networks that are designed to perform in uncertain environments and to maintain connections between elements independent of scale. These properties were originally identified in studies of social networks and the World Wide Web, but they aptly describe the real-world challenges of the cell. Studies of dynamically evolving networks show that nodes (i.e., genes and proteins) added at an early stage (much like highly conserved genes) are more likely to develop many connections, acting as a hub. Following these organizational principles, transcription networks would have a relatively small number of highly connected “hub genes”—though a much higher number than one would expect in a random network.

And that is what the authors observed: the networks they constructed from the expression data had the expected number of highly connected hub genes, which tend to be essential and conserved among organisms. Since these highly connected genes are likely to have homologues in other organisms, they can serve as powerful and efficient tools for assigning function to the thousands of uncharacterized sequences found in sequence databases. This model presents a framework to explore the underlying properties that govern the design and function of the cell and provides important clues—in the form of conserved transcription modules—to the evolutionary building blocks that generate diversity.

**Figure:**
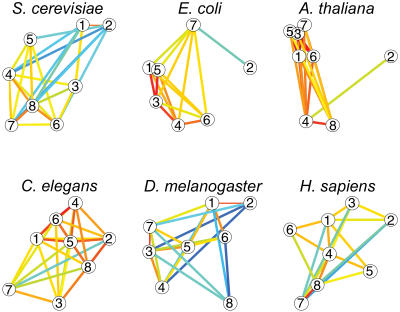
Regulatory relations among transcription modules

